# Research on the influencing factors and mechanism of community education satisfaction

**DOI:** 10.3389/fpsyg.2026.1888377

**Published:** 2026-09-23

**Authors:** Linhui Nie, Shudan Niu

**Affiliations:** 1 Haidian Staff University, Beijing, China; 2 University of Science and Technology, Beijing, China

**Keywords:** chain mediating effect, community education satisfaction, individual needs and participation motivation, sense of community belonging, service quality perception

## Abstract

**Introduction:**

Community education is an important vehicle for building a lifelong learning system for all and a learning society. Scientifically evaluating residents’ satisfaction and the mechanisms by which it is formed is of great significance for improving the quality of community education services. This study examined the factors influencing community education satisfaction and their underlying mechanisms from the perspectives of individual needs and participation motivation, service quality perception, and sense of community belonging.

**Methods:**

A questionnaire survey was conducted among 494 community residents in Beijing. Structural equation modeling with 5,000 bootstrap samples was used to test the proposed chain mediation model, with education level specified as the moderator.

**Results:**

Individual needs and participation motivation, service quality perception, and sense of community belonging all had significant positive direct effects on community education satisfaction. Service quality perception and sense of community belonging also played a chain-mediating role between individual needs and participation motivation and community education satisfaction. Education level moderated only the first-stage path from individual needs and participation motivation to service quality perception, whereas the subsequent path remained stable across groups.

**Discussion:**

This study reveals the chain mediation mechanism underlying community education satisfaction and provides a theoretical reference for community education practice.

## 1 Introduction

Community education is an important vehicle for building a lifelong education system for all and a learning society. It is also the form of education that best embodies educational services spanning all ages and all groups, characterized by full participation, whole-process involvement, and all-round coverage (Shao and Luo, 2026). It plays an important role in meeting the learning needs of all age groups, integrating high-quality educational resources, and ensuring educational equity. Although research on community education remains relatively weak compared with that on higher education, vocational education, and basic education (Song and Xie, 2026), the rapid development of generative artificial intelligence is having a profound impact on education (Yang et al., 2023). In this context, the multi-subject interactive feature of “human–machine collaboration” is shifting from “binary interaction” toward “ternary co-creation” (Li et al., 2025), and a new landscape of community education is worth anticipating. Technological change must ultimately serve people’s needs. Community education should likewise grow organically from residents’ actual needs rather than being delivered solely through top-down design (Wang and Xiang, 2019). A prerequisite for better community education, therefore, is to fully understand residents’ participation in, and satisfaction with, the educational activities currently provided by their communities.

Previous studies have focused on constructing satisfaction models and index systems for community education, typically analyzing the components of satisfaction along dimensions such as organizational management, resource coordination, and effectiveness evaluation (Zhang and Wu, 2020), in an effort to establish a multidimensional and measurable evaluation framework. However, research on the influencing factors of satisfaction has largely examined the independent effects of each factor in isolation; systematic discussion of how these factors are interconnected, how their effects are transmitted, and how they jointly shape overall satisfaction remains limited. To address this gap, this study proposes six research hypotheses (H1–H6; see the Literature Review section for details) and tests them using structural equation modeling (SEM). Specifically, alongside examining the direct effects of the various influencing factors, it tests the chain-mediating transmission mechanism and the moderating effects among these factors, thereby providing new empirical evidence on the formation mechanism of community education satisfaction.

## 2 Literature review and research hypotheses

### 2.1 The influence of individual needs and participation motivation on community education satisfaction

Traditional research on community education satisfaction has mostly been conducted from a supply-side perspective, focusing on the impact of objective service elements—such as infrastructure, teaching staff, and organizational management—on residents’ satisfaction. However, as the “people-oriented” education concept proposed by nine government departments, including the Ministry of Education (2016), has gained ground and residents’ learning needs have become increasingly diverse, researchers have gradually recognized that supply-side factors alone cannot fully explain individual differences in residents’ satisfaction under similar educational supply conditions. Because satisfaction is essentially a subjective psychological evaluation based on individual cognition and experience, individuals differ in how they understand and express satisfaction (Oliver, 1981). Drawing on customer satisfaction theory, Yue (2019) constructed a community education evaluation index system centered on residents’ satisfaction, reflecting a shift from a “supply-oriented” to a “supply–demand interaction” perspective in community education evaluation and treating residents’ subjective feelings as an important basis for evaluation. Research from the perspective of identity theory also shows that residents’ participation in community education is influenced not only by external resource conditions but also by their individual needs, value identification, and participation motivation (Liang and Zheng, 2021). In this context, the role of individual needs and participation motivation—as measurable, guiding internal psychological factors—in shaping community education satisfaction has received increasing attention.

Individual needs and participation motivation generally refer to the psychological characteristics that residents bring to community education participation, such as need type, motivational orientation, and participation expectation. Given the realities of community education practice in China, this study focuses on two dimensions: the degree of need matching and multiple participation motivations. According to expectation–confirmation theory (ECT), satisfaction stems from the degree of confirmation produced by comparing initial expectations with actual experience: satisfaction arises when actual experience meets or exceeds expectations (Bhattacherjee, 2001). ECT further suggests that expectations do not determine satisfaction directly; rather, they exert their influence indirectly by shaping individuals’ perceptions of actual performance. Accordingly, this study treats the individual needs and participation motivation formed before residents’ participation as the psychological basis for expectation formation, and treats service quality perception as residents’ evaluation of actual service performance. Together with satisfaction, these two constructs form the measurable core of the “expectation–perceived performance–satisfaction” transmission chain described by ECT. This study, therefore, focuses on testing the pathway through which the expectation basis in this chain affects satisfaction via perceived performance. In the context of community education, residents’ motivation to participate in educational activities stems from their individual needs, and whether these needs are effectively met during participation directly affects their satisfaction.

In addition to need matching, multiple participation motivations are also important psychological factors affecting community education satisfaction. They refer to the diverse internal driving forces behind residents’ participation in community education activities, including different orientations such as intrinsic interest, social interaction, career development, and external expectations. The Education Participation Scale (EPS) developed by Boshier (1971) is a representative tool for measuring motivation in the field of adult education. Through factor analysis, the EPS identified multiple dimensions of adult learning motivation, including social contact, social stimulation, career development, cognitive interest, community service, and external expectations. Subsequent studies have shown that different types of participation motivation are significantly correlated with satisfaction, but the pathways and strength of their effects vary (You et al., 2016). Research on the motivation of adult learning participation also finds that “interest in learning,” “career development,” “serving society,” and “external expectations” are typical motivation orientations of adult learners in China (Li and Xu, 2004).

Overall, individual needs and participation motivation affect residents’ satisfaction with community education. Although the degree of need matching and multiple participation motivations are conceptually distinct—the former emphasizes the cognitive evaluation of the fit between supply and demand, whereas the latter emphasizes the types of motivation driving participation—the two often operate jointly in actual participation in community education. Therefore, this study integrates them into a single construct, “individual needs and participation motivation,” and systematically examines its impact on community education satisfaction. Based on the above literature, the following hypothesis is proposed:

*Hypothesis 1*: Individual needs and participation motivation affect community education satisfaction.

### 2.2 The influence of service quality perception on community education satisfaction

As an educational service, community education is difficult to assess by a single objective standard. Research on service quality indicates that, for intangible services such as education and consulting, the user’s subjective experience plays an important role in quality evaluation (Oliver, 1981). Building on this, Grönroos (1982) proposed the concept of “perceived service quality,” arguing that service quality does not depend entirely on the objective level of service supply but arises from the perceived gap between the expected service and the actual experience of the service recipient. Subsequently, Parasuraman et al. (1985) proposed the gap model of service quality and further developed the SERVQUAL model, which measures service quality perception along five dimensions: tangibility, reliability, responsiveness, assurance, and empathy (Parasuraman et al., 1988).

In recent years, the SERVQUAL model has been increasingly applied in research on educational services and community education. The perception of service quality is not merely an evaluation of the objective level of service supply but also the result of the interaction between residents’ expectations and their actual experience. From the perspective of residents’ perception of service quality, an evaluation model can be constructed that includes indicators such as tangibility, reliability, responsiveness, assurance, empathy, and the effectiveness of learning resources (Cao and Ding, 2014). Satisfaction with community education is not entirely determined by the supply of educational resources; rather, it is a dynamic psychological evaluation formed during residents’ participation. Moreover, different dimensions influence satisfaction through different mechanisms: some depend more on physical conditions and resource supply, whereas others are more susceptible to residents’ expectation levels, interactive experiences, and depth of participation.

In terms of tangibility, studies have found that the community education site environment, teaching facilities, and hardware conditions constitute an important basis for residents’ overall satisfaction evaluation (Cao, 2015). Well-designed learning spaces and well-equipped teaching facilities can not only improve residents’ direct impressions of education services but also strengthen their perception of the professionalism of community education. In terms of reliability, the stability and standardization of curriculum service delivery are considered important factors affecting residents’ satisfaction. Whether curricula are delivered consistently as scheduled, in the planned locations, and with the planned content, and whether teaching arrangements accommodate residents’ actual life needs, directly affect residents’ trust in community education services (Hu, 2015). In terms of assurance, teachers’ professional competence and teaching attitudes serve as an important basis for community residents—especially elderly learners—to evaluate the quality of education. Research shows that when teachers demonstrate strong professional competence and teach patiently and clearly, residents’ sense of trust and security is significantly enhanced, thereby improving overall satisfaction (Liu et al., 2023). In terms of responsiveness, whether community education institutions can respond to residents’ needs promptly and effectively address problems in the learning process is an important factor affecting the service experience. Studies have shown that the responsiveness of community education organizations to learners’ opinions and suggestions is directly related to residents’ evaluation of the quality of education services (Zhang and Zhang, 2021). In terms of empathy, the degree to which curriculum content matches residents’ actual needs, and whether the teaching format accommodates individually differentiated needs, are important factors affecting satisfaction. Relevant studies have found that when community education better meets the learning needs of different age, occupational, and interest groups, residents’ satisfaction is significantly improved (Wang et al., 2020).

Despite these findings, existing research has mostly focused on constructing index systems for individual service dimensions, and research on how the dimensions of service quality perception jointly affect satisfaction within the overall SERVQUAL framework remains insufficient. Based on the above literature, the following hypothesis is proposed:

*Hypothesis 2*: Residents’ service quality perception affects community education satisfaction.

### 2.3 The influence of sense of community belonging on community education satisfaction

Sense of community is an important concept in community research and social psychology. McMillan and Chavis (1986) proposed that sense of community consists of four dimensions: membership, influence, integration and fulfillment of needs, and shared emotional connection. The sense of community belonging measured in this study focuses on residents’ emotional identification with, and sense of connection to, the community, and is distinct from related concepts such as “social capital,” “community attachment,” and “social cohesion.”

Social identity theory further explains the relationship between the sense of community belonging and satisfaction. Tajfel and Turner (1979) argued that individuals tend to evaluate the groups they identify with more positively in order to maintain a positive self-identity. When residents develop a strong sense of identification with, and belonging to, their community, they are more likely to evaluate community education, community services, and the community environment positively. Kasarda and Janowitz's (1974) study on community attachment also shows that the higher residents’ community attachment, the more positive their evaluations of the overall community environment and public services. Research in China on the sense of community belonging further confirms this view. Based on a survey of Shanghai residents, Zhou (2011) found that urban residents’ sense of community mainly includes two core dimensions: sense of belonging and satisfaction. The two are significantly correlated—the stronger the sense of community belonging, the higher residents’ overall satisfaction with community life. In a study of community governance, Gao and Sun (2021) also argued that sense of community can significantly improve community governance performance, and residents’ satisfaction with community services is one of the important indicators for measuring governance performance. Relevant research shows that the sense of community belonging not only shapes residents’ emotional identification with the community but also influences their evaluation of the quality of community public services.

In the field of community education research, the relationship between the sense of community belonging and community education satisfaction has gradually attracted attention. Zhang et al. (2026), in their study of learning communities, found that members’ sense of belonging is significantly and positively correlated with their well-being and participation experience. The stronger the sense of belonging, the more positive members’ evaluations of community education activities. This shows that community education not only serves the function of knowledge dissemination but also plays an important role in promoting social connection and emotional identification. In addition, some studies have suggested that “running community education that the people are satisfied with” not only means improving the quality of the supply of educational resources but also emphasizes responding to community members’ learning needs, emotional identification, and well-being (Li and Han, 2014). Based on the CCSI model, the study further found that community education participants’ perceived value can enhance their participation loyalty by increasing satisfaction, and satisfaction is closely related to residents’ sense of identity with, and belonging to, the community (Zeng et al., 2024). Taken together, the sense of community belonging is not only an emotional product of participation in community education but also a psychological resource that can, in turn, strengthen residents’ positive evaluations of community education. Based on the above literature, the following hypothesis is proposed:

*Hypothesis 3*: Residents’ sense of community belonging affects community education satisfaction.

### 2.4 The mediating role of perceived service quality

In the context of community education, individual needs and participation motivation may not only directly affect residents’ satisfaction but may also indirectly shape the satisfaction formation process through their perceptions of teaching quality, learning support, and interactive experience. Cronin and Taylor (1992) argued that service quality is an important antecedent of satisfaction and that satisfaction, in turn, affects individuals’ subsequent behavioral intentions, providing a theoretical basis for examining the mediating role of service quality perception in satisfaction formation.

In higher and continuing education, research has increasingly focused on the relationships among learner needs, learning engagement, and service quality perception. Elliott and Shin (2002) argued that student satisfaction derives not only from the supply of educational resources themselves but also from learners’ subjective experience and the fulfillment of individual needs. Sultan and Wong (2013) further found that learners’ perception of education service quality significantly affects their satisfaction and that this perception is typically grounded in holistic experiences such as learning support, interactive experience, and service responsiveness. Al-Sheyadi et al. (2020) argued that education service quality further affects student satisfaction through learners’ overall evaluation of the service process. Yan et al. (2025) further found that learners’ perception of goal attainment can exert a chain mediating effect through variables such as ability acquisition, learning experience, and infrastructure perception, ultimately affecting students’ satisfaction.

Although existing research has examined the mediating role of service quality perception, most studies have focused on higher education or general education services. For community education, the mediating role of service quality perception linking individual needs and participation motivation to community education satisfaction has yet to be examined. Based on the above literature, the following hypothesis is proposed:

*Hypothesis 4*: Service quality perception mediates the relationship between individual needs and participation motivation and community education satisfaction.

### 2.5 The mediating role of sense of community belonging

Residents’ service quality perception may not only directly affect their satisfaction but may also indirectly influence the satisfaction formation process through emotional transformation mechanisms. In this process, the sense of community belonging—an important psychological variable reflecting the emotional connection between residents and their communities—plays an important role in the relationship between cognitive evaluation and satisfaction. Drawing on social exchange theory, Blau (1964) argues that individuals form corresponding attitudes and emotions during social interaction on the basis of the benefits and rewards they receive. When individuals repeatedly gain positive experiences during interaction, they are likely to develop trust, identification, and emotional attachment toward the interaction partner. In the context of community education, when residents perceive that curricula are well designed, teaching support is adequate, and the interactive experience is positive, they not only form a positive evaluation of the education service itself but may also develop stronger emotional identification with, and sense of belonging to, the community. The positive cognitive evaluation arising from the educational service experience may be further transformed into higher satisfaction through emotional identification mechanisms.

Related studies have provided preliminary support for this mechanism. In a study of virtual learning communities, Song et al. (2019) found that the sense of community belonging directly and positively affects user satisfaction and plays an important role in the relationship between learning interaction experience and satisfaction. A positive interactive experience can enhance learners’ sense of belonging to the community, which in turn enhances their overall satisfaction. In addition, the sense of community belonging exerts a strong overall effect among multiple influencing factors, suggesting that it is not only an important outcome of the learning experience but also an important mediating variable in satisfaction formation.

Although existing research has provided initial support for the mediating role of the sense of community belonging between service quality perception and satisfaction, this pathway remains under-examined in the field of community education. Many studies have treated service quality perception and the sense of community belonging merely as parallel predictors of satisfaction, without fully revealing the mediating mechanism between them. Therefore, the following hypothesis is proposed:

*Hypothesis 5*: Sense of community belonging mediates the relationship between service quality perception and community education satisfaction.

### 2.6 Chain mediating effect

In the context of community education, the formation of residents’ satisfaction is not the direct result of a single factor but a dynamic process comprising cognitive evaluation and emotional response. According to self-determination theory, individuals’ internal needs and autonomy affect their behavioral engagement and cognitive processing of the external environment (Deci and Ryan, 1985). When individuals have strong intrinsic motivation, they are more likely to participate actively in learning activities and, in the process, to develop deeper learning experiences and more positive perceptions of service quality. Studies have found that learners’ individual needs and engagement significantly affect their subjective perception of education service quality, and that service quality perception, in turn, affects satisfaction formation (Cronin and Taylor, 1992).

Existing research on learning communities and online learning communities has shown that positive service experiences and interaction quality significantly enhance members’ sense of community, and that a stronger sense of belonging, in turn, affects user satisfaction and willingness to participate (Rovai, 2002). Learners with a strong sense of belonging tend to evaluate the learning environment and educational activities more positively, because a sense of belonging strengthens individuals’ emotional connection to the community and further shapes their overall satisfaction (Sadera et al., 2009).

The above research suggests that a progressive “cognition–emotion” mechanism may underlie the formation of community education satisfaction. Specifically, service quality perception represents residents’ cognitive evaluation of the education service process, whereas the sense of community belonging is the emotional identification formed on the basis of positive service experiences. Residents’ individual needs and participation motivation first influence their evaluation of the education service process; positive service quality perception further enhances their sense of community belonging, and the enhanced sense of belonging ultimately affects overall satisfaction. Although existing studies have verified the relationships between “participation–service quality perception,” “service quality perception–satisfaction,” and “sense of belonging–satisfaction,” systematic discussion of the multivariate continuous transmission mechanism remains relatively limited. It should be noted that this study employs a partial mediation model; that is, the direct path from individual needs and participation motivation to community education satisfaction is retained in the model. Based on the above literature, the following hypothesis is proposed:

*Hypothesis 6*: Individual needs and participation motivation affect community education satisfaction through the chain mediating effect of service quality perception and the sense of community belonging.

The main research relationships of the structural equation model are shown in Figure 1.

**Figure 1:**
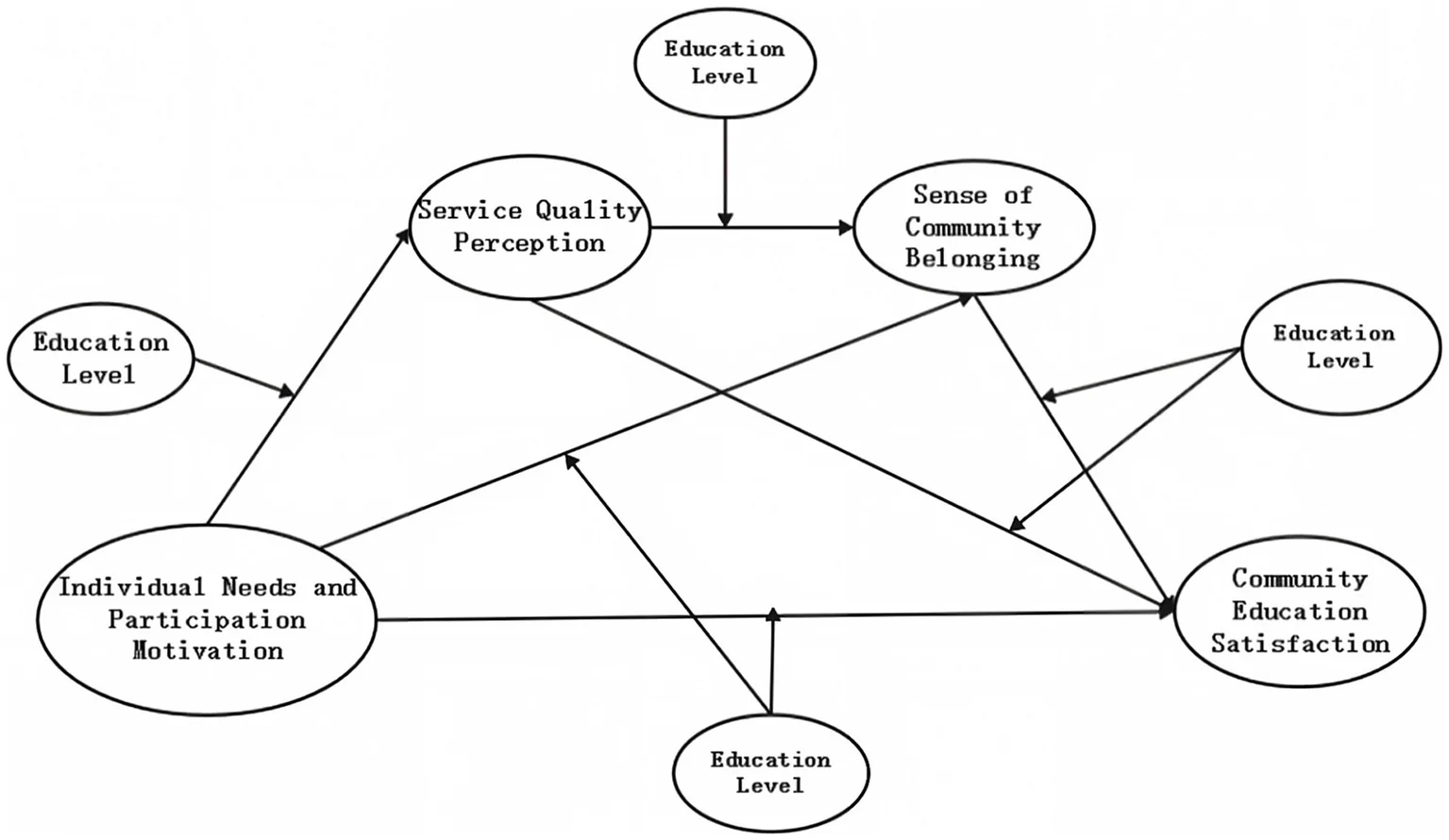
Structural equation model.

## 3 Materials and methods

### 3.1 Participants

This study was an anonymous, low-risk questionnaire survey. Prior to data collection, the research protocol was submitted to and approved by the authors’ institution (Haidian Staff University, Beijing, China), and the study was conducted in accordance with the institutional and national ethical norms for anonymous questionnaire research. Because the institution does not operate an ethics committee approval-number system, this study does not hold an ethics approval number. All participants provided electronic informed consent through the *Questionnaire Star* platform before completing the questionnaire (clicking to begin the questionnaire constituted consent).

This study surveyed 494 residents from different communities in Beijing, China, covering all age groups; the specific distribution is shown in Table 1.

**Table 1:** Distribution of participating community residents.

Age	Number of females	Percentage of females	Number of males	Percentage of males	Total
18–25	12	66.7%	6	33.3%	18
26–35	104	66.7%	52	33.3%	156
36–45	135	69.9%	58	30.1%	193
46–55	54	56.8%	41	43.2%	95
56–65	9	40.9%	13	59.1%	22
65 and older	6	60.0%	4	40.0%	10
Total	320	64.8%	174	35.2%	494

### 3.2 Procedure

The purpose of this study was to examine the relationships among individual needs and participation motivation, service quality perception, sense of community belonging, and community education satisfaction. The respondents were community residents in Beijing. Because community education participants were scattered across various communities and activities were mainly organized through community organization networks, this study recruited participants using convenience sampling: with the assistance of community workers in each district, the researchers forwarded the questionnaire link to community residents via WeChat and invited them to participate voluntarily. The questionnaire did not restrict participation to residents who had previously attended community education activities; any community resident who volunteered could complete it.

The survey was conducted from March 25 to April 2, 2026, via the Questionnaire Star platform, covering many communities in Beijing. To reflect the living patterns of different types of communities in Beijing as much as possible, the distribution process took into account old residential areas, new commercial residential areas, unit communities, urban–rural fringe communities, village-to-residential communities, affordable housing, price-capped housing, low-rent housing, and other major community types. After the questionnaires were collected, the sample involved a total of 15 community types, based on residents’ self-reported community attributes, covering all 16 administrative districts of Beijing. In terms of education level, the sample comprised five respondents with primary school education or below (1.0%), 19 with junior high school (3.8%), 54 with high school/technical secondary school (10.9%), 181 with junior college (36.6%), 213 with a bachelor’s degree (43.1%), and 22 with a master’s degree or above (4.5%). Given that 84.2% of the sample held a college degree or above and the sample sizes of subgroups such as primary school or below, junior high school, and master’s degree or above were too small to support statistical inference independently, education level was recoded into a dichotomous variable in the subsequent moderating effect analysis: high school or below (*n* = 78, 15.8%) and college or above (*n* = 416, 84.2%).

The questionnaires were completed anonymously, and a total of 494 questionnaires were collected. Apart from the inclusion criteria described above, no other exclusion criteria were set. Verification showed that all questionnaires contained complete responses to the questions used in this study; no missing values or completely duplicated response patterns were detected. Therefore, no questionnaires were excluded, and all 494 were included in the analysis. Because the questionnaire was distributed through community WeChat groups, it was impossible to determine the number of people who actually received the link, making it difficult to calculate a response rate in the strict sense. Based on the collected data, structural equation modeling (SEM) was used to analyze the effects of the above factors on community education satisfaction.

In the model analysis stage, a common method variance test was first performed to evaluate potential bias and ensure the validity and reliability of the data. Subsequently, following the standard confirmatory factor analysis (CFA) procedure, the model fit indices were systematically evaluated. After all models met the preliminary fit criteria, service quality perception and sense of community belonging were incorporated into a mediation analysis framework to examine their mediating roles in community education satisfaction.

### 3.3 Measurement tools

Community education satisfaction and its influencing factors were measured using a self-developed questionnaire. In developing the questionnaire, a range of theoretical frameworks were drawn upon, including the Education Participation Scale, the SERVQUAL service quality model, community emotion theory, and the American Customer Satisfaction Index (ACSI) model. The questionnaire items were based on the above theories and adapted to the Chinese community education context; they were neither directly translated nor adapted from existing English scales. To ensure content validity, the items in each dimension were developed strictly in accordance with the construct definitions and operational approaches of the corresponding theories, and construct validity was subsequently tested through confirmatory factor analysis.

The questionnaire first included nine demographic variables—gender, age, education level, length of residence, occupation, frequency of participation, weekly disposable time, and community type—for subsequent control variable analysis and group difference tests. Based on the relevant theoretical frameworks, the questionnaire contained several measurement modules comprising 54 items in total. This study focused on four dimensions—individual needs and participation motivation, service quality perception, sense of community belonging, and community education satisfaction—and selected 29 corresponding items for subsequent analysis. Together with the nine demographic variables above, 38 items were involved in the analysis. It should be noted that the selection of the four dimensions was based on the predefined research framework rather than *post hoc* selection driven by statistical results. All 54 items were retained in the complete questionnaire.

The dimension of individual needs and participation motivation was based on the Education Participation Scale (EPS) developed by Boshier (1971). Through large-scale factor analysis, the scale identified a range of motivational factors, such as cognitive interest, career development, social contact, community service, and external expectations. Subsequent studies further revised the scale, yielding a more stable multi-factor structure. On this basis, this study designed eight items covering need matching, knowledge motivation, skill improvement, and social motivation, so as to capture residents’ internal needs and motivational orientations in community education participation.

The dimensions of service quality perception were based on the SERVQUAL model proposed by Parasuraman et al. (1985, 1988). The SERVQUAL model distinguishes five dimensions of service quality: tangibility, reliability, responsiveness, assurance, and empathy. Based on these five dimensions, this study designed eight items to comprehensively assess residents’ subjective perceptions of the service process and service experience of community education.

The dimension of sense of community belonging was based on the sense of community theory proposed by McMillan and Chavis (1986). The theory identifies four components of sense of community: membership, influence, integration and fulfillment of needs, and shared emotional connection. On this basis, more mature tools for measuring sense of community were developed. Based on this, this study designed five items to measure residents’ emotional attachment to, and psychological identification with, their communities.

The dimension of community education satisfaction was developed with reference to the American Customer Satisfaction Index (ACSI) model. The ACSI model proposed by Fornell et al. (1996) identifies six core variables of satisfaction: customer expectations, perceived quality, perceived value, customer satisfaction, customer complaints, and customer loyalty. The model emphasizes that satisfaction is a comprehensive psychological evaluation formed by consumers on the basis of service experience. Based on this, this study designed eight items to comprehensively evaluate residents’ satisfaction with community education. All items in the four dimensions were rated on a five-point Likert scale, from 1 (strongly disagree) to 5 (strongly agree). In the data collation stage, all items in each dimension were first scored in the positive direction, and the arithmetic mean of the subordinate items in each dimension was then calculated as the final dimension score. The subsequent analyses were based on the mean scores of these four dimensions.

In the moderating effect test, education level was included in the model as a dichotomous variable (0 = high school or below, 1 = college or above), and individual needs and participation motivation were entered using their dimension mean scores. The moderating term was the product of individual needs and participation motivation and the education level dummy variable. Because the moderator was a 0/1 dummy variable, the moderating term had a straightforward interpretation in the two groups (edu = 0 and edu = 1). This study estimated the conditional slopes and bootstrap confidence intervals for the two groups, rather than using the mean ± 1 SD grouping approach. Under this coding scheme, the construction of the moderating term and the estimation of the conditional slopes did not depend on mean centering of the predictor variables.

The overall Cronbach’s α coefficient for the 54 items of the complete questionnaire was 0.919, indicating good internal consistency. For the four dimensions analyzed, the *α* coefficients of the individual needs and participation motivation, service quality perception, sense of community belonging, and community education satisfaction subscales were 0.542, 0.616, 0.559, and 0.649, respectively. It should be noted that the participation motivation dimension covers multiple motivation types, such as knowledge seeking, skill improvement, social interaction, and physical and mental health. The construct has a relatively broad connotation, and the content heterogeneity among its items is relatively high, resulting in a relatively low internal consistency coefficient for this dimension. Overall, the reliability of the questionnaire was considered adequate for the purposes of this study.

Confirmatory factor analysis was conducted with a four-factor model corresponding to four latent variables: individual needs and participation motivation, service quality perception, sense of community belonging, and community education satisfaction. The model fit indices were as follows: *χ*^2^ = 412.158, d*f* = 371, *χ*^2^/d*f* = 1.11, CFI = 0.977, TLI = 0.975, RMSEA = 0.015 (90% CI = 0.000–0.023), and SRMR = 0.035. The standardized factor loadings ranged from 0.205 to 0.497, all significant (*p* < 0.001), which was consistent with the four-factor structure derived from multidimensional theory and supported the construct validity of the questionnaire.

This study further examined the discriminant validity of the four factors. The average variance extracted (AVE) for each factor was as follows: individual needs and participation motivation, 0.134; service quality perception, 0.167; sense of community belonging, 0.204; and community education satisfaction, 0.188. The square roots of the AVE values were 0.366, 0.408, 0.452, and 0.434, respectively. The composite reliability (CR) of each dimension was 0.537, 0.614, 0.560, and 0.649, respectively. The factor correlation matrix showed that individual needs and participation motivation (as a single dimension) and service quality perception were highly correlated at the latent variable level (standardized correlation close to 1), suggesting substantial overlap in their operationalization; the correlations among the remaining factors ranged from 0.207 to 0.258, all lower than the square root of the corresponding AVE, indicating acceptable discriminant validity among the other dimensions. This result is consistent with the theoretical connection between the two: need matching reflects residents’ overall evaluation of whether the supply of community education meets their own needs, and one connotation of service quality perception is the judgment of demand responsiveness. The two concepts overlap. However, at the observed level, the correlation between the two was 0.568, which does not approach complete overlap, and because the subsequent structural equation model was based on dimension mean scores, the high latent-level correlation does not affect the analyses or conclusions of this study. The AVE of each dimension was lower than the recommended threshold of 0.5, reflecting generally low item loadings; convergent validity needs to be strengthened, which is mainly attributable to the high content heterogeneity among items and the broad connotations of the dimension constructs.

To test the robustness of the four-factor structure, the four-factor model was compared with three-factor, two-factor, and single-factor alternative models. The results showed that the fit differences between the four-factor model and the three-factor model (Δ*χ*^2^ = 1.943, Δd*f* = 3, *p* = 0.584), the two-factor model (Δ*χ*^2^ = 6.883, Δd*f* = 5, *p* = 0.229), and the single-factor model (Δ*χ*^2^ = 7.326, Δd*f* = 6, *p* = 0.292) were not statistically significant, consistent with the inter-factor correlations reported above. Because the four-factor structure has a clear theoretical basis and the absolute fit indices reached excellent levels (CFI = 0.977, RMSEA = 0.015, SRMR = 0.035), this study retained the four-factor structure for subsequent analyses.

Considering that individual needs and participation motivation conceptually include two aspects—need matching and participation motivation—this study further compared the single-factor and two-factor models of the eight items in this dimension. The single-factor model fitted well (*χ*^2^ = 27.288, d*f* = 20, CFI = 0.959, TLI = 0.943, RMSEA = 0.027, SRMR = 0.031). In the two-factor model, because need matching was measured by a single item (reflecting residents’ overall cognitive evaluation of the supply–demand fit of community education), the model was unidentified in estimation, making it impossible to obtain stable and reliable fit statistics for model comparison. From the estimable part of the model, need matching was highly correlated with the two participation motivation factors (*r* = 0.745). These results show that separating individual needs and participation motivation into two independent factors lacks measurement support, and that integrating the two into a single construct is empirically acceptable.

### 3.4 Data analysis

First, descriptive statistics were computed for individual needs, participation motivation, service quality perception, sense of community belonging, and community education satisfaction, including the mean and standard deviation of each variable. Bivariate correlations among the variables were then examined using Pearson correlation analysis. In addition, Spearman correlation analysis was used to test the correlation between the moderating variable, education level, and the four variables above. Subsequently, common method variance was evaluated using Harman’s single-factor test. Structural equation modeling was then used to test the chain mediating effect. Specifically, the direct effect path was first tested with individual needs and participation motivation as the independent variables and community education satisfaction as the dependent variable. Service quality perception and sense of community belonging were then included in the model to construct a chain mediation model: individual needs and participation motivation → service quality perception → sense of community belonging → community education satisfaction. All statistical analyses were performed using SPSS 25.0 and Mplus 8.3.

## 4 Results

### 4.1 Common method deviation test

To control for potential common method variance (CMV), two tests were employed in this study. First, Harman’s single-factor test was conducted on the 29 items used in the analysis, following the procedure of Podsakoff et al. (2003). The proportion of variance explained by the first factor was 20.267%, well below the conventional threshold of 50%.

Given the limitations of Harman’s single-factor test, this study further used the Unmeasured Latent Method Construct (ULMC) technique to test this concern: a method factor comprising all 29 items, uncorrelated with the four substantive factors, was added to the four-factor confirmatory factor analysis model, and the model fit before and after adding the method factor was compared. The results showed that, after adding the method factor, the CFI increased from 0.977 to 0.987 (ΔCFI = 0.010), which did not exceed the 0.01 threshold for substantive model differences (Cheung and Rensvold, 2002); although the chi-square difference test was statistically significant (Δ*χ*^2^ = 46.872, Δd*f* = 29, *p* = 0.019), the Chi-square test is sensitive to sample size. Based on the ΔCFI results and Harman’s single-factor test, the contribution of the method factor to model fit was very small, indicating that common method variance did not pose a serious threat to this study.

### 4.2 Descriptive statistics and bivariate analysis

This study constructed a structural equation model to examine the potential effects of education level, individual needs and participation motivation, service quality perception, and sense of community belonging on community education satisfaction. Table 2 presents the means, standard deviations, and correlation matrix of all variables. The descriptive statistics showed that the mean scores of individual needs and participation motivation, service quality perception, sense of community belonging, and community education satisfaction ranged from 3.58 to 3.68, all above the midpoint of the scale. The correlation analysis showed significant positive correlations among individual needs and participation motivation, service quality perception, sense of community belonging, and community education satisfaction, indicating that the three predictor variables had important predictive effects on community education satisfaction and providing a necessary premise for the subsequent mediation analysis. To test for multicollinearity, this study calculated the variance inflation factor (VIF) of each predictor in the structural model; the values ranged from 1.48 to 1.83, indicating no serious multicollinearity among the predictors.

**Table 2:** Means, standard deviations, and correlation matrix of variables.

Variable	*M*	SD	1	2	3	4
1. Individual needs and participation motivation	3.59	0.62	1.000			
2. Service quality perception	3.68	0.66	0.568***	1.000		
3. Sense of community belonging	3.60	0.76	0.529***	0.608***	1.000	
4. Community education satisfaction	3.58	0.69	0.612***	0.658***	0.644***	1.000

*N* = 494; *M*, mean; SD, standard deviation. ***p < 0.001.

To further explore the potential moderating effect of education level, Spearman correlation analysis was used to test the correlations between education level and each variable. The results are presented in Table 3. The results showed that education level was not significantly correlated with individual needs and participation motivation, service quality perception, sense of community belonging, or community education satisfaction. This suggests that education level may function by moderating the relationships between the other variables and satisfaction rather than by directly affecting satisfaction.

**Table 3:** Correlation matrix of education level with various variables.

Variable	1	2	3	4	5
1. Education level	1.000				
2. Individual needs and participation motivation	0.019	1.000			
3. Service quality perception	0.032	0.410**	1.000		
4. Sense of community belonging	0.072	0.417**	0.500**	1.000	
5. Community education satisfaction	−0.106	0.480**	0.503**	0.535**	1.000

*N* = 494. Spearman’s rank correlation coefficients. **p < 0.01.

It should be noted that the absence of significant correlations between the moderating variable and the predictor and outcome variables does not preclude testing its moderating effect. In moderating effect analysis, the focus is on “whether the strength of the relationship between a predictor and the outcome variable changes with the level of the moderating variable,” rather than on the direct correlation between the moderating variable and the outcome variable. Therefore, the non-significant correlations between education level and these variables merely indicate the absence of direct linear relationships with them and do not prevent education level from acting as a moderating variable. To account for the highly skewed distribution of education level, it was recoded into a dichotomous variable (0 = high school or below, *n* = 78; 1 = college or above, *n* = 416) in the moderating effect analysis.

### 4.3 Measurement model verification

This study used structural equation modeling (SEM) to systematically examine how individual needs and participation motivation, service quality perception, and sense of community belonging affect community education satisfaction. First, a structural equation model was constructed with individual needs and participation motivation as the independent variables, community education satisfaction as the dependent variable, and service quality perception and sense of community belonging as mediating variables; the moderating effect of education level was also tested. The results showed that the model had an acceptable fit: *χ*^2^/d*f* = 3.63, CFI = 0.994, TLI = 0.964, RMSEA = 0.073, SRMR = 0.003.

Individual needs and participation motivation had a significant positive predictive effect on community education satisfaction (*β* = 0.271, *p* < 0.001). Service quality perception had a significant positive predictive effect on community education satisfaction (*β* = 0.314, *p* < 0.001). Sense of community belonging had a significant positive predictive effect on community education satisfaction (*β* = 0.310, *p* < 0.001), thus supporting H1, H2, and H3. The interaction between individual needs and participation motivation and education level had no significant direct predictive effect on satisfaction (*b* = −0.199, *p* = 0.068), indicating that education level does not moderate the direct relationship between individual needs and participation motivation and satisfaction.

The moderating effect of education level on the first-stage path from individual needs and participation motivation to service quality perception was further tested. With service quality perception as the dependent variable, Model A included only the main effects of individual needs and participation motivation and education level, whereas Model B additionally included their interaction. Model A explained 32.5% of the variance in service quality perception (*R*^2^ = 0.325). After adding the interaction term, the variance explained by Model B increased to 33.1% (*R*^2^ = 0.331), Δ*R*^2^ = 0.006. The unstandardized regression coefficient of the interaction term was 0.275 (SE = 0.127, *p* = 0.031), and the bias-corrected 95% confidence interval obtained from 5,000 bootstrap samples was [0.032, 0.538], excluding 0. The conditional slope analysis showed that, in the high school or below group, individual needs and participation motivation had a significant positive predictive effect on service quality perception (*b* = 0.355, SE = 0.118, *p* = 0.003); in the college or above group, this effect was stronger (*b* = 0.629, SE = 0.050, *p* < 0.001), indicating that education level positively moderates the path from individual needs and participation motivation to service quality perception. It should be noted that the contribution of the interaction term to *R*^2^ was relatively small (Δ*R*^2^ = 0.006), which is common in research on moderating effects. The evidence for the moderating effect mainly comes from the interaction coefficient and its bootstrap interval, as well as the pattern of differences between the two conditional slopes.

To investigate the mediating roles of service quality perception and sense of community belonging, this study estimated the effect, standard error, and bias-corrected 95% confidence interval of each indirect path based on 5,000 bootstrap samples. The indirect effect of the path “individual needs and participation motivation → service quality perception → community education satisfaction” was 0.202 (SE = 0.033, 95% CI [0.141, 0.272]). The indirect effect of the path “individual needs and participation motivation → sense of community belonging → community education satisfaction” was 0.093 (SE = 0.020, 95% CI [0.060, 0.140]); the chain indirect effect of the path “individual needs and participation motivation → service quality perception → sense of community belonging → community education satisfaction” was 0.089 (SE = 0.016, 95% CI [0.062, 0.128]). None of the confidence intervals for the three indirect paths contained 0. The total indirect effect was 0.384 (SE = 0.040, 95% CI [0.311, 0.466]), the direct effect (*c*′) was 0.301, and the total effect (*c*) was 0.684. The chain indirect effect accounted for 13.0% of the total effect (95% CI [9.0, 18.4%]).

To test the plausibility of the chain structure, this study further constructed a parallel mediation model (without the path from service quality perception to sense of community belonging) and a reverse chain model (with the order of the two mediators reversed) for comparison. The fit of the parallel model deteriorated substantially (*χ*^2^ = 106.568, d*f* = 1, RMSEA = 0.462, CFI = 0.879). Compared with the chain model, the difference was significant (Δ*χ*^2^ = 106.568, Δd*f* = 1, *p* < 0.001), indicating that model fit decreased significantly after removing the chain path from service quality perception to sense of community belonging. Although the two parallel indirect paths in the parallel model were significant (0.202 and 0.182; 95% CI [0.133, 0.247]), the overall fit of the parallel model was inferior to that of the chain model, indicating that the independent roles of the two mediators could not replace the chain transmission between them. The reverse chain model and the forward chain model were statistically equivalent (both saturated), so their fit indices could not be distinguished directly. The chain indirect effect was 0.080 (95% CI [0.052, 0.116]), slightly lower than that of the forward chain path (0.089). The total indirect effects of the three models were consistent (all 0.384), indicating internal consistency of the estimates. Combined with the theoretical framework, the chain order—service quality perception first, followed by the sense of community belonging—is more consistent with the causal logic of the SERVQUAL and sense of community theories. Therefore, this study retained the forward chain model as the final model; the results are shown in Figure 2.

**Figure 2:**
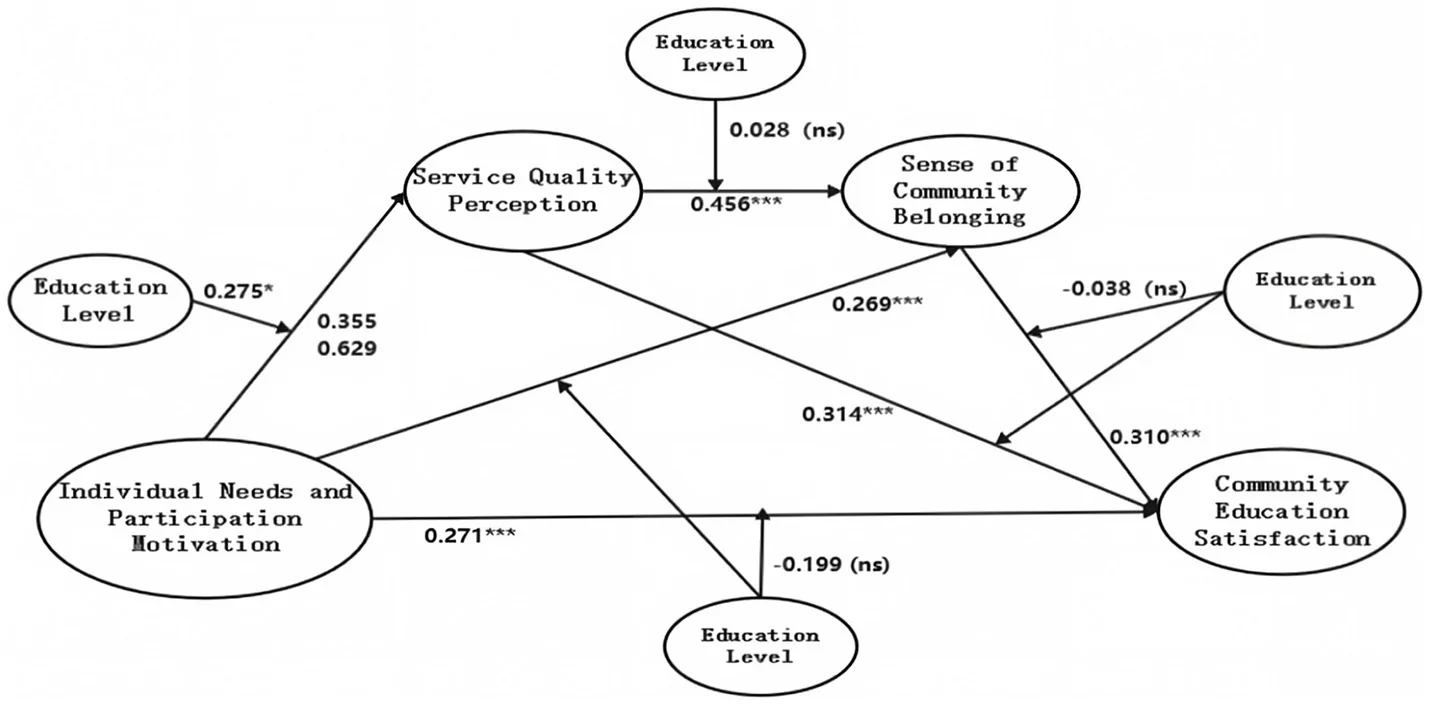
Structural equation modeling results. Path diagram of the chain mediation model with educational attainment as moderator. Standardized coefficients are shown; *p < 0.05, ***p < 0.001; ns, not significant. Dashed arrows denote interaction effects (unstandardized *b*): educational attainment moderated the path from individual needs to perceived service quality (*b* = 0.275*) but not the direct path to satisfaction (*b* = −0.199, ns). Coefficients on the individual needs–perceived service quality path are simple slopes for the two education groups.

The hypothesis testing results of the structural equation model were as follows: H1, H2, H3, H4, H5, and H6 were all supported. In addition, the moderating effect of education level on the path from individual needs and participation motivation to service quality perception was supported (*b* = 0.275, *p* = 0.031), whereas its’ moderating effects on the direct path from individual needs and participation motivation to satisfaction and on the other mediating paths were not supported.

## 5 Discussion

Based on empirical data from 494 community residents, this study constructed a model with service quality perception and sense of community belonging as chain mediating variables and education level as the moderating variable. The results confirmed that individual needs and participation motivation not only directly affect community education satisfaction but also indirectly affect it through the chain transmission of service quality perception and sense of community belonging, and that the first stage of this process is moderated by education level.

### 5.1 The direct impact of each variable on community education satisfaction

The results showed that individual needs and participation motivation, service quality perception, and sense of community belonging each had a significant positive predictive effect on community education satisfaction. Individual needs and participation motivation had a significant positive predictive effect on satisfaction (*β* = 0.271, *p* < 0.001), supporting H1. From the perspective of expectation–confirmation theory, residents’ curiosity, skill improvement, or social expectations when participating in community education constitute a set of psychological benchmarks for evaluating their educational experience. When course content responds to these needs, expectations are confirmed and satisfaction arises (Bhattacherjee, 2001). Service quality perception had a significant positive predictive effect on satisfaction (*β* = 0.314, *p* < 0.001), supporting H2 and indicating that residents’ actual experiences of venues and facilities, teacher competence, service responsiveness, and other aspects are closely related to their overall evaluation of community education. Sense of community belonging had a significant positive predictive effect on satisfaction (*β* = 0.310, *p* < 0.001), supporting H3. This result shows that community education satisfaction is not purely a service evaluation but is also related to the emotional connection between residents and their communities. When individuals perceive that community resources meet their own needs, their sense of belonging and satisfaction are higher. This result is consistent with the theoretical expectation that the sense of community belonging, as an emotional identification, affects overall evaluation. These results are consistent with previous research on educational services: the positive predictive effect of service quality perception on learning satisfaction has been verified in higher education (Elliott and Shin, 2002). And the positive correlation between sense of community belonging and educational satisfaction is also consistent with findings from research on virtual learning communities (Song et al., 2019).

In addition, among the three direct effects, the path coefficient of individual needs and participation motivation (0.271) was relatively low, lower than those of service quality perception (0.314) and sense of community belonging (0.310). This suggests that clear participation needs alone are not sufficient: the relationship between needs and satisfaction may be mediated by actual service experience and emotional identification.

### 5.2 The mediating effect of service quality perception and sense of community belonging

The study further explored the internal mechanism through which individual needs and participation motivation affect community education satisfaction. H4, H5, and H6 proposed that individual needs and participation motivation predict service quality perception and sense of community belonging, and that these two variables play a chain mediating role between individual needs and participation motivation and satisfaction. All three hypotheses were supported. Individual needs and participation motivation had a significant positive predictive effect on service quality perception (*β* = 0.568, *p* < 0.001). Service quality perception had a significant positive predictive effect on sense of community belonging (*β* = 0.455, *p* < 0.001), and the chain mediating effect was significant.

In terms of effect sizes, the total effect of individual needs and participation motivation on community education satisfaction was 0.684, of which the direct effect was 0.301 and the indirect effect was 0.384, accounting for 56.1% of the total effect. Among the three indirect paths, the path via service quality perception was 0.202, the path via sense of community belonging was 0.093, and the chain path was 0.089; the chain path accounted for 13.0% of the total effect. This result is consistent with the hypothesized indirect path model, indicating that the relationship between individual needs and participation motivation and satisfaction is largely realized through the mediating roles of service quality perception and sense of community belonging.

There was a significant positive correlation between individual needs and participation motivation and service quality perception, indicating that participation motivation is closely related to evaluations of the service experience. According to expectation–confirmation theory, individuals form initial expectations based on their own needs before receiving a service. These expectations guide individuals to attend to and process information during the service process (Bhattacherjee, 2001). In this study, residents with clear learning needs and strong participation motivation entered community education in a pre-activated psychological state. This state may encourage them to pay more attention to the practicality of course content. Evaluate teachers’ professional competence more carefully, and seek service responsiveness more actively during participation, thereby obtaining more positive evaluations of the service experience under otherwise identical service conditions. It should be noted that this explanatory path is inferred from expectation–confirmation theory. This study did not directly measure the processes of expectation formation and information processing; this mechanism should be further tested in future research.

These results are consistent with the progressive “cognition–emotion” mechanism: residents first form a cognitive evaluation of the service process, then develop an emotional identification with the community on this basis, which in turn affects their overall satisfaction. However, given the limitations of the cross-sectional design, the specific ordering of this step-by-step process needs to be confirmed with longitudinal data.

### 5.3 The moderating effect of education level

It should be noted that the moderating effect of education level was an exploratory finding identified during the data analysis, rather than a confirmatory test based on prior hypotheses. The results showed that education level moderated only the first-stage path from individual needs and participation motivation to service quality perception (*b* = 0.275, *p* = 0.031) and had no significant moderating effect on the subsequent paths or the direct effect of the mediating chain. The interaction term had no significant predictive effect on community education satisfaction (*b* = −0.199, *p* = 0.068). In addition, education level had no significant direct effect on sense of community belonging (*b* = 0.060, *p* = 0.433) or community education satisfaction (*b* = −0.072, *p* = 0.154). This indicates that the moderating effect of education level is mainly reflected in the relationship between individual needs and participation motivation and service quality perception. In the college or above group, the positive association between individual needs and participation motivation and service quality perception (*b* = 0.629) was stronger than that in the high school or below group (*b* = 0.355), indicating that the strength of this association varies with education level. One possible explanation for this pattern is that residents with higher education levels may hold clearer evaluation criteria for quality factors such as course depth and teacher competence, and may therefore perceive differences in service quality more accurately; residents with lower education levels may lack the corresponding reference standards, even when they have a strong willingness to participate.

One possible reason the moderating effect was confined to the first-stage path is that the transformation from individual needs and participation motivation to service experience perception involves residents’ active cognitive processing, which is more sensitive to the evaluative frame of reference and therefore more susceptible to the influence of education level. Once the service experience is formed, its transformation into a sense of community belonging and satisfaction depends more on emotional processing, which shows a high degree of consistency across different cultural groups. In addition, other possible explanations exist. For example, the measurement items for service quality perception and sense of community belonging may be interpreted differently by groups with different education levels; or the high school or below group was small (*n* = 78), so statistical power was limited, which may have weakened the detection of moderating effects on the subsequent paths. Future research could distinguish among these alternative explanations by expanding the sample and conducting measurement invariance tests.

In addition, education level did not moderate the path from service quality perception to sense of community belonging, nor the path from sense of community belonging to satisfaction. This indicates that the relationships between service quality perception and sense of community belonging, and between sense of community belonging and satisfaction, remained stable across groups with different education levels. Therefore, community education should attend to the needs and perception characteristics of residents with different education levels at the course provision stage and provide more targeted services, whereas a relatively unified strategy may be adopted in cultivating community emotions.

## 6 Limitations and future directions

This study has several limitations. First, the sample was drawn from a single region, the sample size (*N* = 494) was relatively limited, and residents were recruited through convenience sampling. Caution is therefore needed in generalizing the findings to other regions, other types of communities, and broader population groups. In addition, this study did not collect data from residents who did not participate in community education, so the sample may be biased toward community education participants. The demographic variables collected were limited and did not include socioeconomic characteristics such as income level, occupational status, and marital status, which may be related to residents’ participation motivation and satisfaction evaluations. Moreover, the study adopted a cross-sectional design, so the temporal sequence among variables could not be established. The mediation and moderation results should therefore be interpreted as statistical associations compatible with the hypothesized model, rather than as evidence of causal mechanisms. Future research could use longitudinal data to further test the causal direction among variables.

Second, all variables were measured through residents’ self-report questionnaires, so the influence of common method variance (CMV) cannot be completely ruled out. Although the Harman single-factor test and the unmeasured latent method construct (ULMC) approach were used for diagnosis, the self-report data may still be affected by social desirability bias, and residents may tend to report satisfaction more positively. In addition, this study did not include objective service indicators such as course provision and facilities, so residents’ subjective perceptions could not be compared with the actual level of supply. Although this study drew on the analytical framework of expectation–confirmation theory, it did not directly measure the degree of confirmation between expectations and actual performance. Future research could combine evaluations from community education administrators, objective participation data, and a complete measure of expectation confirmation to further enhance the robustness of the conclusions.

In addition, the measurement instrument of this study was a self-developed questionnaire. Although its development was guided by theory and its structure was supported by confirmatory factor analysis, it was not subjected to expert review or a pilot test. The internal consistency coefficients of the dimensions were relatively low. Individual needs, participation motivation, and service quality perception were highly correlated at the latent variable level, leaving room for improvement in the quality of scale measurement. The verification of the factor structure and the common method variance test were both based on the same sample, which was not randomly divided into exploration and validation subsamples, limiting the robustness of the testing strategy. Future research could improve the measurement by optimizing item wording and expanding the sample.

Finally, the moderating effect of education level was an exploratory finding confined to the first-stage path of the mediating chain. The increment in R^2^ attributable to the interaction term was relatively small (Δ*R*^2^ = 0.006), so the relevant conclusions should be interpreted with caution. The moderating effects on the subsequent paths were not significant. Whether other, more explanatory moderating variables exist (e.g.), age, length of residence, or community type) requires further investigation. Future research could collect larger samples, incorporate more complete demographic information, and adopt a longitudinal tracking design to reveal the dynamic changes and causal mechanisms underlying the formation of community education satisfaction.

## 7 Conclusion

This study surveyed 494 community residents and systematically examined the mechanisms through which individual needs and participation motivation, service quality perception, and sense of community belonging influence community education satisfaction by constructing a chain mediation model. The results showed that all three variables had significant positive direct effects on satisfaction, with perceived service quality showing the strongest predictive effect; service quality perception and sense of community belonging played a chain mediating role between individual needs and participation motivation and satisfaction; and education level moderated only the effect of individual needs and participation motivation on service quality perception, with no significant moderating effect on the subsequent paths, indicating that, once the service experience is formed, its transformation into sense of community belonging and satisfaction is stable across groups with different education levels. These findings reveal the chain transmission mechanism of community education satisfaction and provide a theoretical reference for community education practice.

## Data Availability

The original contributions presented in the study are included in the article/supplementary material, further inquiries can be directed to the corresponding author.
